# GIDISdb: a gene expression database for exploring human immune responses in infectious diseases

**DOI:** 10.1093/database/baag036

**Published:** 2026-07-09

**Authors:** Yu Liao, Zhen-Lin Tan, Wen-Kang Shen, Wei Li, Qian Lei, An-Yuan Guo

**Affiliations:** Department of Laboratory Medicine, West China Biomedical Big Data Center, West China Hospital, Sichuan University, Chengdu, 610041, China; Department of Laboratory Medicine, West China Biomedical Big Data Center, West China Hospital, Sichuan University, Chengdu, 610041, China; Hubei Key Laboratory of Bioinformatics and Molecular-Imaging, College of Life Science and Technology, Huazhong University of Science and Technology, Wuhan, 430074, Hubei, China; Department of Laboratory Medicine, West China Biomedical Big Data Center, West China Hospital, Sichuan University, Chengdu, 610041, China; Hubei Key Laboratory of Bioinformatics and Molecular-Imaging, College of Life Science and Technology, Huazhong University of Science and Technology, Wuhan, 430074, Hubei, China; Hubei Key Laboratory of Bioinformatics and Molecular-Imaging, College of Life Science and Technology, Huazhong University of Science and Technology, Wuhan, 430074, Hubei, China; Department of Laboratory Medicine, West China Biomedical Big Data Center, West China Hospital, Sichuan University, Chengdu, 610041, China; Department of Laboratory Medicine, West China Biomedical Big Data Center, West China Hospital, Sichuan University, Chengdu, 610041, China

## Abstract

Decoding human immune responses to infectious diseases through transcriptomic analysis is crucial for understanding disease progression and guiding therapeutic development. The absence of a comprehensive database integrating infectious disease transcriptomes with immune-focused analyses limits such efforts. Here, we developed the GIDISdb (a Gene expression database for Infectious DISeases) to address this. Distinct from existing resources that focus on single pathogen types or lack standardized analytical workflows, GIDISdb uniquely integrates cross-disease transcriptomic data with an immune-centric analytical framework. GIDISdb integrates 3949 whole-blood RNA-seq samples from 51 projects across 15 types of bacterial, viral, and fungal infections (e.g. AIDS, COVID-19, and tuberculosis). By leveraging this extensive collection, GIDISdb enables detailed transcriptome analysis, including differential expression analysis and gene set enrichment analysis, for various diseases and their subgroups. Beyond traditional transcriptome analysis, it integrates immune-specific annotations from ImmPort, Reactome, and gene ontology, encompassing genes and pathways related to immune function, as well as estimated immune cell abundance. In a case study comparing latent tuberculosis (LTBI), active tuberculosis (TB), and healthy controls, LTBI showed upregulated microbial defense genes and downregulated inflammatory genes relative to controls, along with reduced IFN–γ signaling compared to active TB. Immune-cell profiling revealed partially restored dendritic cells and expanded regulatory T cells, indicating a balanced immunoregulatory network. In summary, GIDISdb is a comprehensive, immune-centric platform for investigating gene expression in infection immunology, poised to accelerate the discovery of infection-specific immune mechanisms and biomarkers. The GIDISdb is accessible at https://guolab.wchscu.cn/GIDISdb/.

## Introduction

Infectious diseases result from complex interactions between pathogenic microorganisms and human immune responses, in which inadequate or dysregulated immune responses often determine clinical outcomes [1]. While systemic infections, such as sepsis account for substantial global mortality [2], the molecular mechanisms underlying host–pathogen interactions remain incompletely characterized. Transcriptomic profiling has emerged as a powerful approach to decode these mechanisms, revealing how immune responses are orchestrated at the transcriptional level during infections [3]. However, the lack of integrated large-scale transcriptomic data with clinical context across diverse infections hinders these findings.

Recent advances in bioinformatics have yielded specialized databases for infectious disease research. DualSeqDB provides dual RNA sequencing data that capture simultaneous transcriptome changes in seven bacterial pathogens and their hosts during infection [4]. MVIP focuses on viral infections, integrating multiomics data to map comprehensive virus–host interaction networks [5]. Wu *et al.* [6] established the Flu-CED, which integrated 8064 samples from 103 independent datasets collected from GEO and ArrayExpress. Chew *et al.* [7] generated a large–scale, multicentre whole–blood transcriptome dataset from 502 patients with diverse respiratory infections, including COVID–19, influenza, sepsis, and coinfections. While these resources offer valuable pathogen-specific insights, several important limitations remain. Existing databases generally lack (i) cross-disease integration enabling direct comparison of immune responses across bacterial, viral, and fungal infections, (ii) user-friendly, immune–centric workflows for bulk RNA–seq data analysis—users need no coding or bioinformatics expertise, and (iii) interactive immune pathway visualizations with different groups, subset, and filter functions for exploring e.g. natural killer (NK) cytotoxicity, B cell receptor (BCR) signalling, and interferon response.

To bridge this gap, we developed GIDISdb, a curated resource that systematically integrates RNA-seq data of 3949 clinically annotated patient RNA-seq samples from 51 project datasets across 15 types of bacterial, viral, and fungal infections. GIDISdb provides precomputed analytical outputs through a unified framework. It enables intradisease comparisons (e.g. Patients vs. Healthy; Severe vs. Mild cohorts) and cross-disease analyses to identify conserved or pathogen-specific immune signatures. All data are harmonized via standardized pipelines with immune-functional annotations, allowing researchers to directly explore differentially expressed genes (DEG), pathway enrichment, and immune cell abundance through interactive queries. Together, these features establish GIDISdb as a comprehensive, immune-centric platform for cross-disease investigation of host transcriptomic responses in infectious diseases.

## Materials and methods

### Data collection and preprocessing

Data of healthy controls and subjects with infection-related diseases were collected through the following process. The retrieval of raw data involved sourcing SRA or fastq files from NCBI Sequence Read Archive and EMBL-EBI European Nucleotide Archive databases using search terms tailored to each group, including ‘healthy control whole blood RNA-seq’ and ‘(infection disease name) whole blood RNA-seq’. The samples were required to be whole blood samples from nonpregnant individuals of Homo species, categorized as healthy controls or patients with infection-related diseases. Sample-level inclusion criteria required that each sample had complete clinical metadata (including disease status, age, and sex where available), while exclusion criteria included samples with ambiguous infection status, or insufficient sequencing depth (<10 million reads) or an alignment rate below 70% during RNA–seq quantification. To ensure data uniformity and facilitate integrative analysis, microarray data were also excluded.

The 3949 harmonized human whole/peripheral blood RNA-seq samples included in GIDISdb were derived from 51 curated transcriptomic BioProject datasets. These datasets encompass diverse infectious diseases across more than 15 microbial infection categories, including AIDS, tuberculosis (TB), COVID-19, influenza, enterovirus/rhinovirus, dengue virus, metapneumovirus, candidemia, bacterial pneumonia, seasonal coronavirus, bacterial infections (including predominant pathogens such as *Streptococcus pneumoniae* and *Staphylococcus aureus*, along with *Escherichia coli, Klebsiella pneumoniae, Haemophilus influenzae*, and polymicrobial infections), sepsis, and viral infections (e.g. HIV–TB, coinfections). The GIDISdb further stratifies cases by severity, treatment regimens, and clinical outcomes provided by metadata. The datasets distribution and cohort metadata are detailed in Table 1.

**Table 1:** The summary of meta information of GIDISdb.

Group	Number of samples
AIDS	369
Bacterial (*Streptococcus pneumoniae, Staphylococcus aureus*, polymicrobial, *Escherichia coli, Haemophilus influenza, Klebsiella pneumoniae, Legionella* sp., *Streptococcus agalactiae, Streptococcus pyogenes*, and *Proteus mirabilis*)	92
Bacterial pneumonia	24
COVID-19	656
Candidemia	211
DNA virus (adenovirus, cytomegalo virus, Ebstein–Barr virus, and herpes simplex virus)	16
Dengue virus	21
Enterovirus or rhinovirus	31
Healthy	876
Influenza	82
Metapneumovirus	17
Mixed candida/bacterial	79
Respiratory RNA virus (parainfluenza virus and respiratory syncytial virus)	17
SIRS (not infection)	34
Seasonal Coronavirus	61
Sepsis	550
Tuberculosis	696
Virus (influenza A and B, rhinovirus, respiratory syncytial virus, human metapneumovirus, Coronavirus, Coxsackievirus/echovirus, rhinovirus, and parainfluenza)	117
Total	3949

SIRS: systemic inflammatory response syndrome.

All data processing was performed using standardized software versions to ensure reproducibility: HISAT2 (version 2.2.1) was used for read alignment; Samtools (version 1.15) for SAM/BAM conversion and sorting; FeatureCounts (from Subread package version 2.0.3) for gene expression quantification; and R (version 4.2.0) with Bioconductor (version 3.15) for downstream statistical analyses. The alignment of transcriptome data was performed using HISAT2 [8]. The conversion and sorting of SAM to BAM formats were completed using Samtools [9]. Following alignment, FeatureCounts was employed for the final stage of gene expression quantification, utilizing GENCODE v31 annotations based on the GRCh38.p13 genome annotation file [10].

### DEGs and gene set enrichment analysis analysis

Within each infectious disease cohort dataset, we processed the raw gene expression data by removing genes with low expression (defined as a sum of raw read counts <50 across all samples) and retaining only protein-coding genes for further analysis. Raw read counts were then transformed to transcripts per kilobase per million (TPM) to standardize expression levels across samples. To focus on biologically relevant signals, we applied rigorous filtering criteria. Genes with a TPM mean value <1.0 across samples were removed, effectively eliminating genes with insignificant sequencing results. For cross-dataset DEG analysis integrating multiple datasets into disease-specific cohorts (e.g. influenza vs. AIDS), batch effects were corrected using the ComBat algorithm from the sva package (version 3.44.0), with batch defined by the original BioProject accession of each dataset [11]. Differential expression analysis was performed using DESeq2 (version 1.36.0), applying a negative binomial model to identify DEGs [12]. To address multiple testing, *P*-values were adjusted using the false discovery rate (FDR) method. The selection of DEG thresholds was guided by both statistical and biological considerations: an FDR ≤ 0.05 was chosen as the standard for multiple-testing correction, while |log2FC| ≥ 1 corresponds to a minimum two-fold expression change, which is widely accepted as biologically meaningful in transcriptomic studies. The additional filtering criteria requiring mean TPM > 1 in at least one comparison group ensures that retained genes are expressed at detectable levels, thereby reducing false positives from low-expression noise.

After finishing the DEG analysis, we further analysed gene functional enrichment. We performed gene set enrichment analysis (GSEA) based on gene sets from gene ontology (GO, *n* = 10 447), Kyoto Encyclopedia of Genes and Genomes (KEGG, *n* = 186), and Reactome (*n* = 1 615) function-annotated gene sets. We calculated GSEA results by clusterprofiler based on DEGs identified in the previous step [13]. To ensure the accuracy of enrichment results, only those gene sets with *P*-adjusted value/FDR ≤ .05 were considered significant.

### WGCNA analysis

Weighted gene coexpression network analysis (WGCNA) was performed using the R package WGCNA (version 1.71) to identify coexpressed gene modules associated with the case–control phenotype. Prior to analysis, genes with mean TPM > 1 in at least one group were retained, and the expression matrix was normalized with the voom function from the limma package to obtain log2–counts per million (log2–CPM) values; genes expressed in fewer than 20% of samples were additionally excluded. WGCNA was carried out only for comparison groups in which both case and control groups had >15 samples each.

Sample clustering was first performed to detect and visualize potential outliers. The soft–thresholding power was determined using pickSoftThreshold to achieve a scale–free topology fit (*R*² > 0.85); if no power met this criterion, a default power of 6 was used. A signed coexpression network was then constructed with the blockwiseModules function, applying a signed network type, a minimum module size of 30, and a mergeCutHeight of 0.25.

Module eigengenes were calculated and correlated with the case/control trait to identify phenotype–associated modules. For each gene, gene significance (GS) for the case trait and module membership (MM) were computed, and hub genes within modules were defined as those with |GS| > 0.2 and |MM| > 0.8.

Modules showing significant correlation with the case group (|r| > 0.3 and *P* < .05) were selected for functional enrichment analysis. For each selected module, the number of genes was evaluated to determine the appropriate input: modules with fewer than 50 genes were skipped; for modules containing 50–500 genes, all genes were used; for modules with more than 500 genes, only hub genes (|GS| > 0.2 and |MM| > 0.8) were used. Modules with fewer than five genes available for analysis were also excluded. Enrichment analyses were then performed for KEGG pathways, Reactome pathways, and GO terms (biological process, cellular component, and molecular function), and only significantly enriched terms (*P* < .05) were retained and reported.

### Immune cell abundance calculation

To evaluate immune cell composition from RNA-seq data in different groups, we utilized ImmuCellAI (Immune Cell Abundance Identifier; https://github.com/lydiaMyr/ImmuCellAI) to quantify the relative abundances of 24 immune cell subtypes [14]. Differential abundance analysis between two experimental groups was performed using the Mann–Whitney U test for pairwise comparisons. Statistical significance was defined as *P*-values ≤ .05.

### Immune function genes annotation

Immune gene annotation was performed to categorize genes and pathways relevant to infectious disease responses (e.g. influenza, SARS-CoV-2). Curated gene lists from the ImmPort Shared Data repository (https://www.immport.org/shared/genelists) were used, integrating both legacy and updated versions to ensure comprehensive coverage of immune-related genes. These resulted in 3545 immune-related genes and 482 immunological pathways. DEGs and GSEA results were annotated for immune functions by mapping to these ImmPort-derived genes and pathways.

### Construction of database

The GIDISdb database web interface was developed using Vue.js (https://vuejs.org/) and Element Plus (https://element-plus.org) for the frontend, with a Flask (https://flask.palletsprojects.com/en/3.0.x) backend and MongoDB (https://www.mongodb.com/) for data storage. Interactive visualizations were implemented using ECharts (https://echarts.apache.org).

## Results

### Overview of GIDISdb architecture and data composition

**Figure 1:**
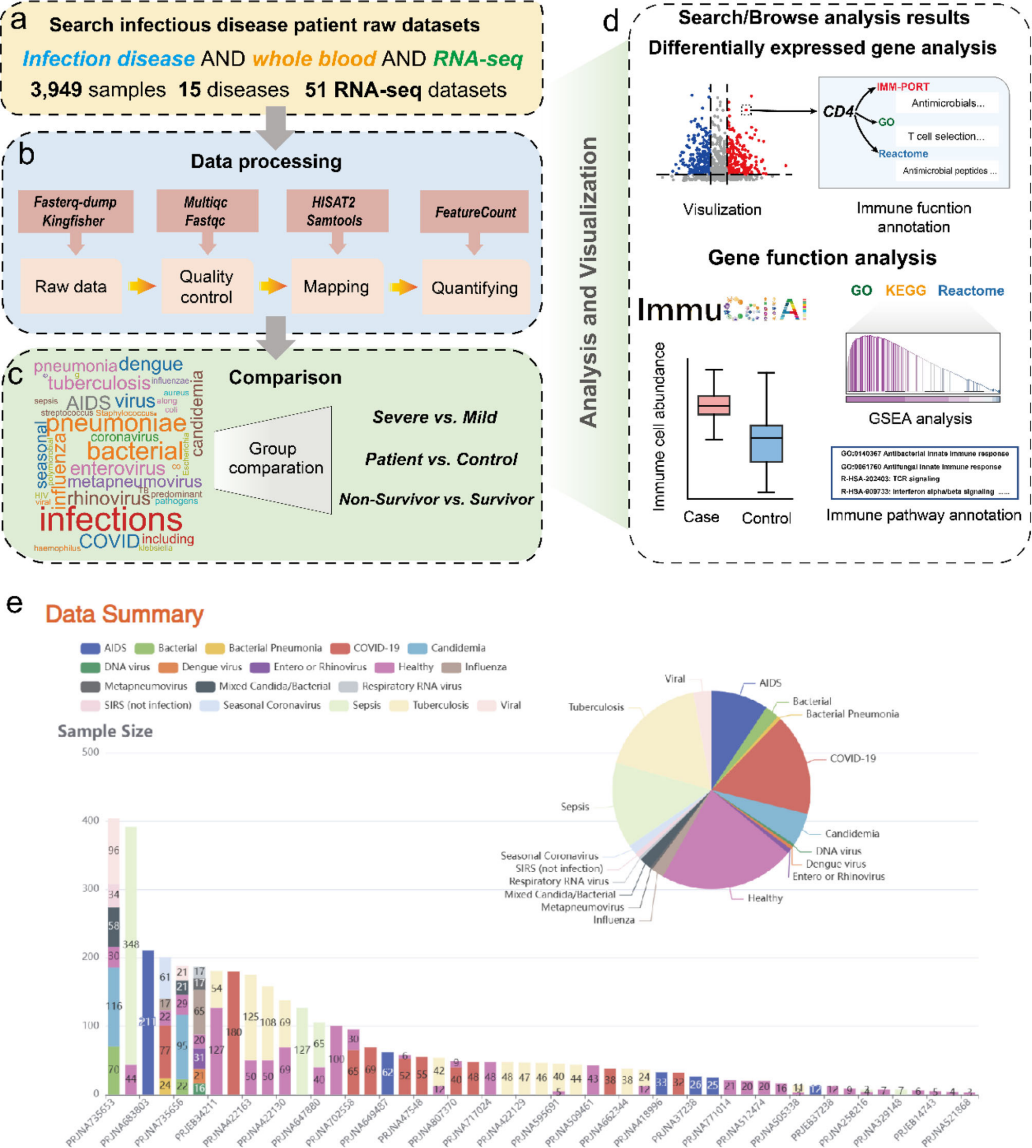
The overview of GIDISdb. GIDISdb workflow: (a) Retrieve raw RNA-seq data from whole blood of infectious disease patients using open-access repositories (search query: ‘Infection disease’ AND whole blood AND RNA-seq). (b) Generate standardized expression matrices through uniform processing pipelines. (c) Stratify patients into clinical subgroups (e.g. severe vs. mild, nonsurvivors vs. survivors) and diseases using group information from original metadata. (d) Core analytical modules: DEGs analysis with immune functional annotation, pathway enrichment GSEA analysis results with immune-related mechanisms pathway filter and ImmuCellAI-quantified abundance of 24 immune cell types. (e) Overview of the diseases represented and their corresponding projects included in the dataset [17]. DEG: differentially expressed gene.

GIDISdb provides a comprehensive transcriptomic database for investigating human immune responses in infectious diseases, integrating 3949 human whole-blood RNA-seq samples from 51 BioProjects. The dataset spans 15 clinically relevant infection types, including bacterial (e.g. TB and streptococcal pneumonia), viral (e.g. COVID-19 and influenza), and fungal (e.g. candidemia) pathogens (Fig. 1a and e; Table 1). All datasets were uniformly processed through a standardized analytical pipeline, including quality control, mapping, and expression quantification (Fig. 1b). The samples are annotated with detailed clinical metadata (pathogen type, disease severity, and outcomes) to enable stratified comparisons between infection states (e.g. infected vs. control) and clinical subgroups (e.g. severe vs. mild and nonsurvivors vs. survivors) (Fig. 1c).

The database integrates analytical results, including DEGs (FDR ≤ 0.05, |log_2_FC| ≥1 and mean TPM > 1 in one group) and functional enrichment terms across ImmPort, GO, KEGG and Reactome databases. Within the functional enrichment results, immune-specific pathways (e.g. cytokine signalling and phagocytosis regulation) are highlighted in a visualization module (Fig. 1d). All outputs are presented through both static publication-ready figures and dynamically interactive visualizations, with export functionality for all data types. This integrated framework delivers analysis-ready transcriptomic and clinical data through an intuitive web interface, facilitating rapid exploration of infection mechanisms. GIDISdb stands out from competitors like HIHISIV, SysInflam HuDB, RNA2Immune, and ImmPort with support for ≥ 15 infectious diseases, precomputed DEG/GSEA results, searchable immune genes/pathways, and simulated immune-cell abundances from bulk RNA-seq, features often absent or partial in others. The comparison results are in Table 2.

**Table 2:** Feature comparison of GIDISdb and other immune-related databases.

Feature	GIDISdb	Hihisiv	SysInflam HuDB	Rna2immune	ImmPort	Flu‐CED
Multiple infectious diseases (≥15)	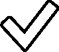	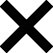 (HIV only)	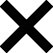 (inflammation)	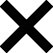 (ncRNA)	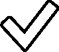 (broad)	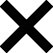 (Influenza)
Precomputed DEG results	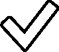	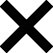	Partial (only tabular DEG results)	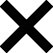	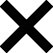	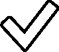
Precomputed GSEA results	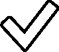	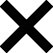	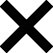	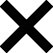	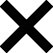	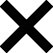
Search for both immune genes and pathways in precomputed results	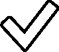	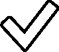	Partial	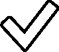	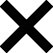	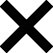
Results of simulated immune-cell abundances from bulk RNA-seq	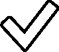	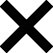	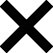	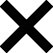	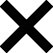	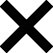

### Functions and features of GIDISdb

#### Browse module for dataset-level immune gene exploration

**Figure 2:**
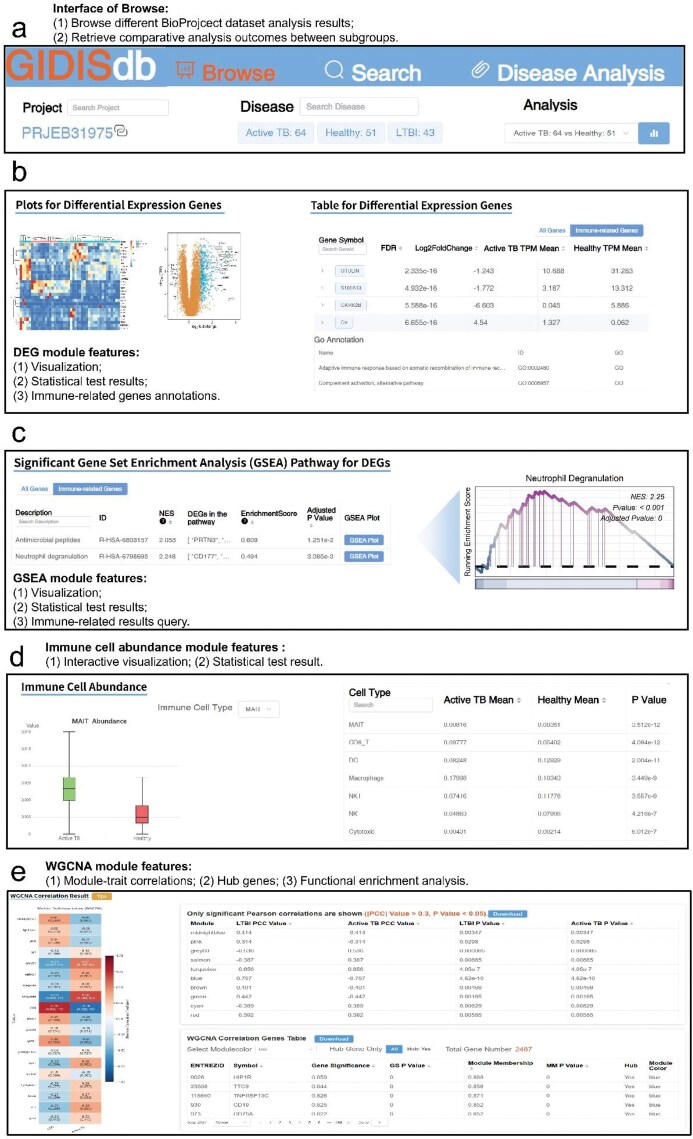
The ‘Browse’ module of GIDISdb. (a) Main interface of ‘Browse’ module, for dataset queries by BioProject ID or disease name, supporting various subgroup comparisons. (b) DEGs analysis results for selected cohorts, displaying immune function annotations by GO, Reactome, and ImmPort. (c) GSEA enrichment of pathways from KEGG, Reactome, and GO derived from DEGs, and highlight immune-related pathways from Reactome and GO. (d) Interactive analysis of immune cell abundances, showing boxplots for 24 immune cell types as calculated by ImmuCellAI, and the significance of Mann–Whitney U tests between the two selected groups. FDR: false discovery rate. NES: normalized enrichment score. (e) WGCNA analytical functions, including module-trait correlation distributions, hub gene identification from key phenotype-relevant modules, and functional enrichment analysis of pivotal modules.

The ‘Browse’ module supports dataset-level exploration of gene expression patterns stratified by key clinical factors (e.g. disease severity and infection type) using preprocessed datasets. Users can select preprocessed datasets by BioProject or disease name for comparative analyses (e.g. case vs. control) (Fig. 2a). Differential expression results are displayed as heatmaps and volcano plots, and also show in Tables with each DEG’s log_2_FC, FDR, and mean TPM in each comparison group (Fig. 2b). Users can also selectively examine the subset of DEGs associated with immune functions.

Functional annotation of DEGs is carried out via GSEA based on pathways/functions from GO, KEGG, Reactome, and ImmPort. Significantly enriched pathways are classified as up– or downregulated based on normalized enrichment scores (NES) and FDR values. Each significant GSEA result is visualized through a detailed enrichment plot that highlights the DEGs driving the enrichment by tables (Fig. 2c). Immune–associated pathways identified by GSEA are presented separately for focused investigation. For cell-level analysis, the module integrates ImmuCellAI to infer the abundance of 24 immune cell types per sample, enabling immune microenvironment characterization (Fig. 2d). Gene coexpression network analysis is a powerful approach to uncover the intrinsic regulatory relationships of genes and mine phenotype-associated core gene clusters. Therefore, WGCNA was performed on all expressed genes with TPM values greater than 1 across samples. This workflow included module-trait correlation distributions, identification of hub genes from key phenotype-relevant modules, and functional enrichment analyses of pivotal modules, aiming to dissect gene expression regulatory networks and identify key functional genes linked to the research phenotype (Fig. 2e). All outputs, including Tables and Figs., are downloadable.

#### Search module for immune gene and pathway interrogation

**Figure 3:**
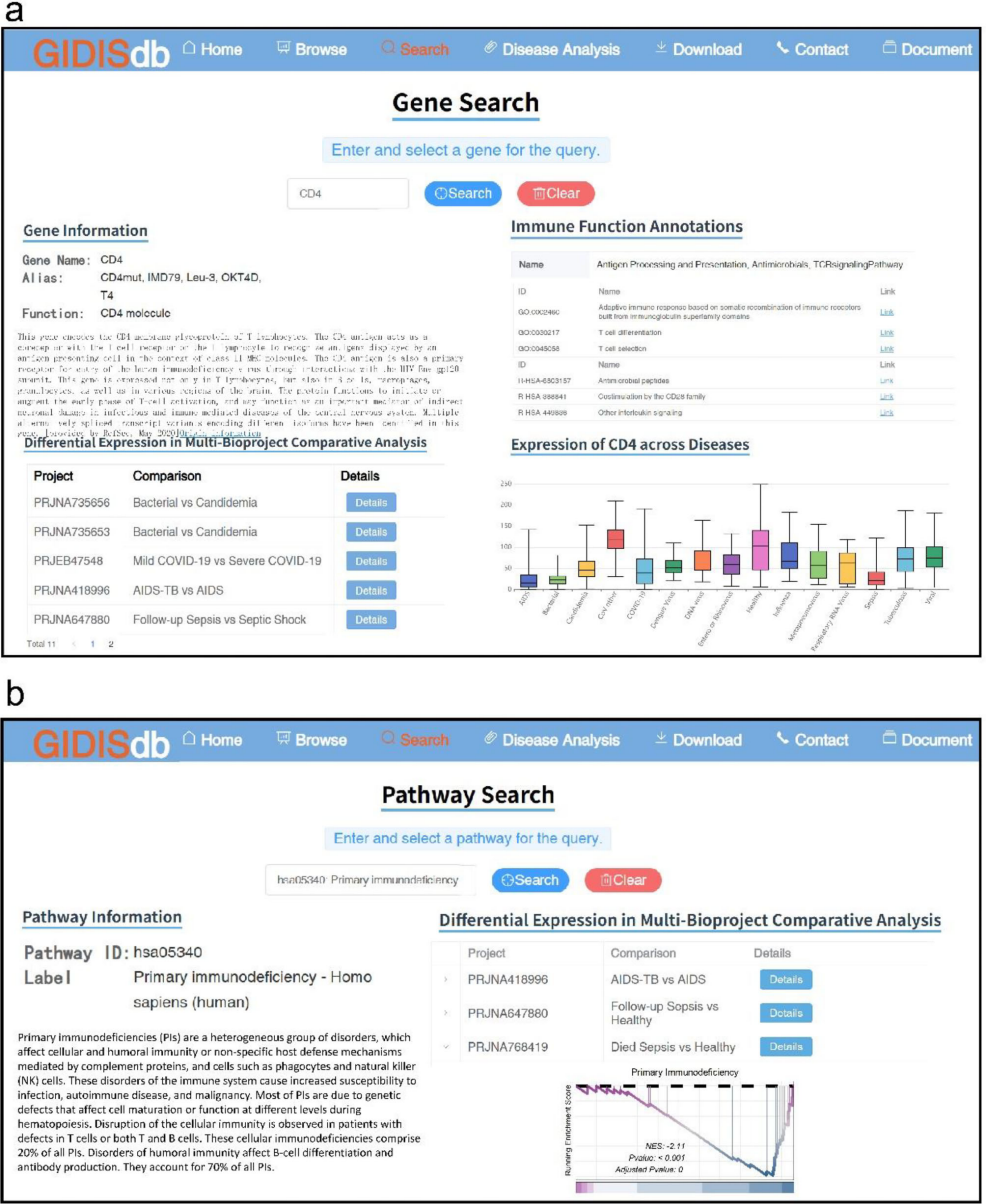
The ‘Search’ module of GIDISdb. (a) Features of ‘Gene Search’ submodule: (1) Gene functional annotations of the target gene (NCBI, top left); (2) Immune function annotation of the target gene (top right); (3) Differentially expressed status of the gene (FDR ≤ 0.05, |log_2_FC|≥1) across BioProject subgroups comparisons (bottom left); (4) TPM expression profiles of the target gene across different disease (bottom right). (b) Features of pathway search submodule: (1) Functional annotation and description of the target pathways in source databases (left); (2) Statistically enriched pathways (FDR/*P*-adjusted value ≤ .05) across different group comparisons, with available detailed views of each enrichment results (right). FDR: false discovery rate. TPM: transcripts per million.

The ‘Search’ module enables rapid, hypothesis-driven interrogation of gene- and pathway-level mechanisms across heterogeneous infection datasets. Gene Search: Users query any gene (e.g. CD4, IL6, or nonimmune genes like GAPDH) to retrieve: (1) functional annotations, including immune-related pathway membership (e.g. ‘cytokine signalling’ for CD4 from ImmPort, Reactome, or GO). (2) A summary table highlights BioProject subgroups and infectious disease cohorts (such as influenza vs. SARS-CoV-2 or AIDS vs. healthy controls) showing significant differential expression (FDR ≤ 0.05) (Fig. 3a). (3) Interactive barplots and boxplots display log_2_FC and expression levels (TPM), respectively, for each queried gene across all 15 infectious diseases (Fig. 3a). These visualizations enable users to explore conserved or divergent roles of genes, such as CD4 or IL6, across patient cohorts with distinct infection groups (e.g. AIDS or healthy controls). This facilitates mechanistic insights into infection outcomes, such as immune dysregulation in AIDS or viral persistence in chronic HIV infection.

Pathway Search: Users query pathway identifiers (e.g. Reactome ID: R-HSA-168256) or functional keywords (e.g. ‘interferon signalling’) to retrieve pathway-level results (Fig. 3b). Each entry provides the pathway’s source database (KEGG, Reactome, or GO), identifier, functional description (e.g. ‘Type I interferon signalling pathway’ for antiviral responses), and enrichment status. A summary table highlights BioProject subgroups and infectious disease cohorts (such as influenza or AIDS), showing significant pathway enrichment (FDR ≤ 0.05). Detailed enrichment plots for each GSEA result allow direct comparison of NES and FDR values across significantly enriched groups (Fig. 3b). This enables identification of key mechanisms, such as dysregulated interferon signaling in severe COVID-19 versus healthy controls.

#### Disease analysis module for cross-disease immune signature discovery

**Figure 4:**
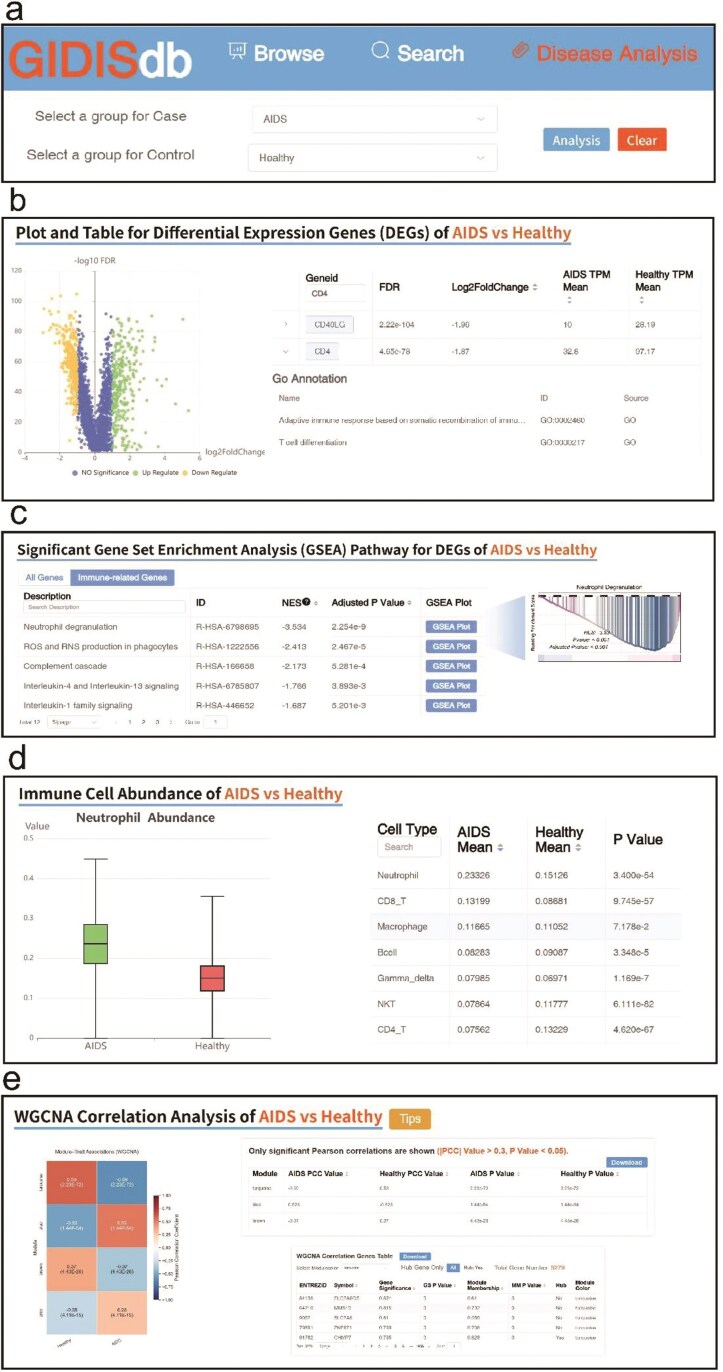
The ‘Disease Analysis’ module of GIDISdb. (a) Interface for defining comparative analyses by selecting a reference disease and a target disease. (b) Interactive volcano plot visualizing differentially DEGs with searchable tables linking genes to immune functions (e.g. antigen processing, inflammatory response). (c) GSEA of significantly enriched pathways (KEGG, Reactome, and GO) and immune-specific pathways (Reactome and GO). (d) Interactive analysis of immune cell abundances, showing boxplots for 24 immune cell types as calculated by ImmuCellAI, and the significance of Mann–Whitney U tests between the two selected groups. FDR: false discovery rate. NES: normalized enrichment score. DEG: differentially expressed gene. (e) WGCNA analytical functions, including module–trait correlation distributions, hub gene identification from key phenotype-relevant modules, and functional enrichment analysis of pivotal modules.

The ‘Disease Analysis’ module enables researchers to explore transcriptomic profiles across integrated, harmonized multicohort datasets, categorized by pathogen class (e.g. viral and bacterial) and disease type (Fig. 4a). Through systematic comparisons of these transcriptomic profiles aggregated from large-scale patient cohorts, the module facilitates identifying conserved or pathogen-specific immune mechanisms. An intuitive interface allows users to flexibly define comparison pairs (e.g. COVID-19 vs. sepsis), supporting bidirectional analyses to reveal both shared and divergent immune signatures. For each user-defined disease pair, the platform delivers multidimensional immunological insights via the following components:

1. Differential expression analysis: Immune-related DEGs are annotated using functional terms from ImmPort (e.g. ‘cytokine activity’), Reactome (e.g. ‘Interferon Signalling’), and GO (e.g. ‘innate immune response’) (Fig. 4b). Interactive volcano plots allow users to visualize expression changes for specific genes (e.g. CD4 or IL6) between disease conditions (e.g. AIDS vs. healthy controls), revealing immune-related mechanisms underlying infection outcomes.
2. Pathway enrichment analysis: GSEA results evaluate differential activation of pathways (e.g. ‘T-cell activation’ and ‘Type I interferon signalling’) across disease comparisons (Fig. 4c). Interactive enrichment plots display NES and FDR ≤ 0.05, categorized into up- and downregulated pathways, enabling exploration of immune pathway dynamics (e.g. enhanced interferon responses in SARS-CoV-2 infection).
3. Immune cell deconvolution: Estimated immune cell abundance is inferred via computational deconvolution and visualized through interactive boxplots, allowing comparison of microenvironmental shifts across disease states (Fig. 4d). These plots highlight changes in immune cell populations (e.g. increased CD8+ T cells in AIDS), facilitating insights into immune alterations driving infection outcomes.
4. WGCNA analysis: Interactive visualizations present module–trait correlation patterns and core hub genes within key functional modules, followed by module-based pathway enrichment analyses to characterize the biological functions of pivotal gene clusters (Fig. 4e). This systematic network analysis uncovers latent gene regulatory mechanisms underlying phenotypic variations and pinpoints critical hub genes (e.g. CHMP7) driving disease-related molecular alterations.

All visual outputs are interactive and exportable, and DEG-level annotations are hyperlinked to external resources such as GeneCards for extended functional insights. To enhance usability, we also provide a prefiltered subset of immune-related DEGs and GSEA results, allowing focused exploration of immunologically relevant genes. The module thus supports cross-disease immunological comparisons that can reveal both shared host defense strategies and pathogen-specific immune evasion mechanisms.

### Case study

#### Decoding immune signatures in latent tuberculosis infection

Latent tuberculosis infection (LTBI) represents a clinically asymptomatic state in which individuals harbor *Mycobacterium tuberculosis* (MTB) without manifesting symptoms. Current diagnostics, such as tuberculin skin tests and interferon-gamma release assays (IGRA), show limited sensitivity in distinguishing LTBI from healthy individuals [15].

**Figure 5:**
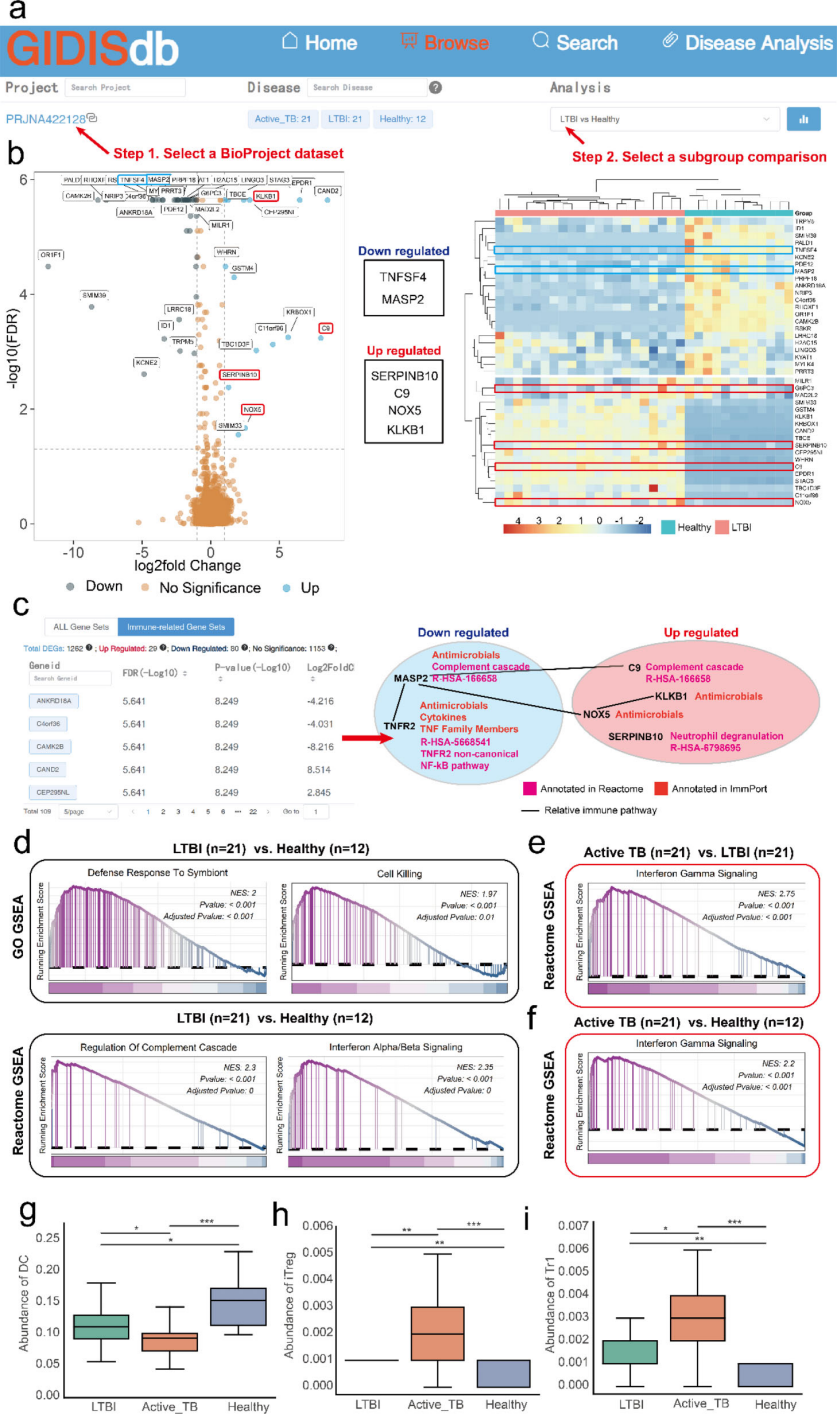
Analysis of latent tuberculosis patients with healthy individuals. (a) The LTBI vs. healthy comparison analysis results by selecting PRJNA422128 dataset in ‘Browse’ module. (b) Volcano plot and heatmap of DEGs (FDR ≤ 0.05, |log_2_FC|≥1), highlighting immune-related DEGs. (c) Immune genes, interactions, and functional annotations derived from GIDISdb-curated immune DEGs. (d) Significantly enriched immune-related pathways (FDR ≤ 0.0) from GO and Reactome. (e and f) Reactome Interferon gamma signalling pathway (R-HSA-877300) in active TB vs. LTBI/healthy (active TB vs. healthy, NES = 2.2, FDR < 0.001; active TB vs. LTBI, NES = 2.75, FDR < 0.001). (g–i) Deconvoluted immune-cell abundances from bulk RNA-seq in LTBI, active TB, and healthy groups: (g) Dendritic cells (DCs), (h) iTreg, and (i) Tr1 cells. Statistical significance was determined by the Mann–Whitney U test. *, FDR < 0.05; **, FDR < 0.01; ***, FDR < 0.001. TB: Tuberculosis. LTBI: latent infection tuberculosis. NES: normalized enrichment score. FDR: false discovery rate.

To investigate gene-level immune features in LTBI, we applied GIDISdb to analyze the PRJNA422128 dataset (active TB, LTBI, and healthy cohorts; Fig. 5a). Differential expression analysis between LTBI and healthy controls identified multiple immune-related DEGs: TNFSF4 and MASP2 were significantly downregulated, whereas SERPINB10, C9, NOX5, and KLKB1 were upregulated (Fig. 5b and c). The upregulated genes (e.g. SERPINB10 and C9) were enriched in pathways associated with microbial defense, consistent with the observed suppression of MTB proliferation in LTBI patients. And downregulation of MASP2 (a key component of the complement lectin pathway) and TNFSF4 (involved in T-cell activation) was observed in LTBI, suggesting attenuated inflammatory responses that may limit inflammation and lung damage—consistent with the asymptomatic clinical of LTBI. However, reduced expression of these genes could also reflect impaired immune control over MTB, aligning with the risk of progression from LTBI turn into active TB in some individuals [15,16]. Notably, interferon-γ (IFN-γ)-associated pathways—an essential biomarker to TB infection 15—were not significantly enriched in LTBI. However, active TB patients from the same cohort showed robust IFN–γ pathway activation compared to both LTBI and healthy controls (Fig. 5f and g), in line with their symptomatic disease and positive IGRA. The absence of IFN–γ enrichment in LTBI thus offers one plausible explanation for false–negative IGRA outcomes.

Building on the immune signatures observed in LTBI, we next examined the immune-cell abundances results in database. In LTBI, dendritic cells (DCs) abundances displayed an intermediate proportion—higher than active TB (but lower than healthy controls; Fig. 5g)—indicating partial restoration of antigen-presenting capacity compared to the suppressed state in active disease [17]. Simultaneously, iTreg abundances were significantly elevated in LTBI versus healthy individuals (Fig. 5h), yet reduced compared to active TB patients. Tr1 cells showed a similar pattern (Fig. 5i). This profile reflects a finely tuned immunoregulatory network in LTBI: moderately recovered DC function supports controlled antigen exposure, while expanded Tr1 and iTreg populations actively dampen IFN-γ-driven inflammation—establishing a nonpathogenic, contained infection state that limits tissue damage but sustains latent viability of MTB, underlying the risk of reactivation [17–19].

## Discussion

GIDISdb provides a centralized platform for analyzing 3949 clinical samples across 15 infectious diseases, addressing the gap in accessible and standardized infectious disease transcriptomic data. By applying a unified preprocessing pipeline, it ensures reproducibility and enables immune-centric gene and pathway annotations that allow researchers to rapidly query disease-specific transcriptomic immune signatures. This functionality directly supports downstream applications, such as biomarker discovery and hypothesis generation for experimental validation.

GIDISdb harmonizes diverse datasets spanning bacterial, viral, and fungal infections, enabling cross-pathogen comparisons rarely achievable in individual studies. By integrating ImmPort, Reactome, and GO immune pathway annotation, the database prioritizes immune function relevant biological pathways, streamlining hypothesis generation in infection immunology. With its interactive modules (Browse, Search, and Disease Analysis) cater to diverse research needs—from single-gene queries to multidisease gene pathway enrichment analysis—while interactive visualizations simplify interpretation of complex results. The case study on latent tuberculosis demonstrates GIDISdb’s capacity to resolve subtle immune signatures, offering immunity mechanism explanations for diagnostic challenges like IGRA false negatives in LTBI patients. In the LTBI case study, GIDISdb’s results reveal downregulation of TNFSF4 and MASP2, suggesting compromised cellular and innate immunity that may blunt IFN–γ release in vitro, thus contributing to IGRA false negatives [15]. These immune signatures are further reflected in immune-cell abundance patterns in LTBI. DCs show intermediate levels—higher than in active TB but lower than in healthy controls. Meanwhile, iTreg and Tr1 subsets are elevated relative to healthy individuals but reduced compared to active TB. This pattern suggests a partial restoration of antigen-presenting capacity accompanied by controlled immunoregulation, which may limit tissue damage while maintaining latent MTB viability, offering mechanistic insight into the subclinical containment observed in LTBI [17–19]. By detecting these subtle transcriptomic shifts, GIDISdb not only offers mechanistic insights into diagnostic gaps but also guides the refinement of biomarkers and testing strategies.

Compared to specialized resources, such as HIHISIV, SysInflam HuDB, and RNA2Immune [20–22], GIDISdb uniquely integrates various infections, by harmonizing these transcriptomes with clinical metadata, GIDISdb enables stratified differential expression and enrichment analyses that other databases cannot. Built–in immune annotations, interactive visualizations, and unified data export streamline workflows. The ‘Disease Analysis’ module allows direct cross–infection comparisons of precomputed DEG sets, helping researchers identify shared and pathogen–specific immune signatures on one platform.

While GIDISdb currently focuses on whole or peripheral blood transcriptomes, this choice is supported by the accessibility of blood as systemic immune readout, as also reflected in resources like ImmuneSigDB [23, 24]. However, several limitations remain. First, the exclusive reliance on whole/peripheral blood data restricts insights into tissue- or cell type-specific immune dynamics. Future versions could address this by integrating tissue-resolved datasets (e.g. lung biopsies in TB). Second, although GIDISdb currently includes 50 BioProject datasets, the rapid growth of infection genomics demands ongoing updates; implementing an automated pipeline for real-time data ingestion would improve sustainability. Additionally, host factors such as comorbidities, genetic background, and drug interactions remain under annotated due to inconsistent metadata availability and privacy constraints. This gap limits investigations into how host heterogeneity influences infection outcomes. Finally, while pathogen categories (viral/bacterial/fungal) are annotated, strain-level details (e.g. SARS-CoV-2 variants and drug-resistant MTB) are sparse, hindering analyses of genotype–phenotype relationships. Addressing these limitations through expanded metadata curation and pathogen genomic integration will enhance GIDISdb’s utility for precision infectious disease research.

As part of our ongoing commitment to keeping the database current and comprehensive, we have established a clear future update plan. The updates will focus on incorporating newly published RNA-seq datasets to substantially expand the current collection. In addition, we will enhance the platform’s analytical capabilities by introducing dedicated modules for biomarker query and analysis. To ensure full transparency and reproducibility, we will implement a robust data version management system. To ensure full transparency and reproducibility, a robust data version management system will be implemented, with each update assigned a unique version number (e.g. GIDISdb v1.1) and detailed release notes. We intend to perform major updates every year and minor updates on a quarterly basis. All historical versions will remain accessible to users.

In conclusion, GIDISdb addresses a major challenge in infectious disease research by systematically integrating discrete transcriptomic datasets into a standardized analysis platform. Through this detailed transcriptomic database, users can intuitively explore biological mechanisms—particularly those related to immune function—across different patient populations and disease severities via interactive tables and visualizations. By providing open access to harmonized RNA-seq data and analysis results, GIDISdb supports collaborative efforts to decode human immune mechanism against pathogens and accelerate translational outcomes.

## Supplementary Material

baag036_Supplementary_Tables

## Data Availability

All datasets involved in this project are summarized in Table S1, which provides the BioProject accession numbers, release dates, corresponding institutions, PubMed references, and main contributors.
